# Early life high-fat diet exposure evokes normal weight obesity

**DOI:** 10.1186/s12986-020-00464-w

**Published:** 2020-06-24

**Authors:** Yuko Maejima, Shoko Yokota, Shoichiro Horita, Kenju Shimomura

**Affiliations:** Department of Bioregulation and Pharmacological Medicine, Fukushima Medical University School of Medicine, 1 Hikarigaoka, Fukushima City, Fukushima, 960-1295 Japan

**Keywords:** Juvenile, High fat diet, Normal weight obesity, Rat, Model

## Abstract

Obesity is becoming one of the most severe global health problems. However, risk of developing normal weight obesity, where an individual has a high percentage of body fat despite a normal body mass index, is gaining attention since such individuals also develop systemic inflammation and metabolic dysregulation.

In this study, juvenile (3-week-old) and adult (8-week-old) rats were fed a high fat diet (HFD) for 9 weeks and compared them with normal chow diet (NCD) fed rats. The HFD fed adult group showed increase in energy intake, body weight (BW), total fat, visceral fat and subcutaneous fat compared with an age-matched NCD group. In addition, the percentage of muscle mass to BW in the adult HFD group was significantly lower compared with the NCD group. When HFD feeding was started from the juvenile stage, there were almost no differences in energy intake and BW between the HFD and NCD groups. However, the juvenile HFD group showed a 1.7-fold increase in total fat, visceral fat and subcutaneous fat compared with their age-matched NCD group. The percentage of muscle mass to BW was significantly lower in the juvenile HFD group compared with the NCD group. In addition, increased plasma insulin levels and decreased insulin sensitivity was observed only in juvenile HFD group, but not in adult HFD group. These results suggest that HFD feeding in growth period induces insulin resistance and normal weight obesity.

Here we show a method for generating a normal weight obesity model, as well as raising the alarm for developing normal weight obesity when children are exposed to high-fat meals.

## Introduction

The prevalence of obesity is one of the most severe health concerns worldwide [1]. “Obesity” is defined as body mass index (BMI) greater than or equal to 30 according to the World Health Organization classification, with abnormal or excessive fat accumulation that may impair health. Because of its simplicity, BMI has been widely used for validation of obesity in multiple epidemiologic studies. However, recent studies have revealed the high prevalence of individuals with normal weight obesity [2–4], which is defined as individuals with normal weight and normal BMI but with high fat percentage (> 20% in men and > 30% in women) [2]. Individuals with normal weight obesity are known to present with dysregulated metabolism, and are therefore at high risk of developing metabolic syndrome and cardiometabolic dysfunction; such individuals also have a higher mortality rate [5, 6].

The modern lifestyle is characterized by a lack of physical activity and excessive energy intake from high caloric diet in both children and adults. In the juvenile stage, animals, including human, are in a dramatic growth period and have very different energy metabolism and feeding regulations compared to adult animals [7]. In our previous study, we showed that neurons in the ventral tegmental area that regulate feeding and energy metabolism show undeveloped properties in weaning rats [7]. Thus, it can be considered that juvenile animals, which are in a dramatic growth period, are easily influenced by the balance of nutrition.

Previous reports revealed high fat diet (HFD) on post-weaning stage affects mental and physiological functions in juvenile animals. Post-weaning HFD consumption increased anxiety behavior via decreasing serotonergic immunoreactivity in macaques [8] or resulted in lesions of growth cartilage with increasing plasma levels in markers of chronic inflammation [9]. Acute exposure to HFD impairs hippocampal function in a glucocorticoid receptors dependent manner in juvenile rats [10]. These reports suggested that HFD feeding in juvenile animals results in dysfunction of brain and bones. However, there are no reports that examined the effects related to metabolic syndrome when juvenile animals were exposed to HFD. We hypothesized that juvenile exposure to HFD may promote more severe state of obesity and metabolic syndrome compared with adult exposure to HFD.

In the current study, we aimed to examine the effects of a HFD on energy intake and, fat and glucose metabolism at an early stage of life. The data of the present study indicate the larger effect of HFD on energy metabolism at early stage and may induce normal weight obesity. This study provides the method for generating a normal weight obesity model, and suggests the importance of assessing not only BMI but also the level of fat accumulation when evaluating one’s health status.

## Material and methods

### Animals

Male Wistar rats, aged 3 weeks (juvenile) and 8 weeks (adult) were purchased from Japan SLC (Shizuoka, Japan).

The animals were maintained on a 12-h light/dark cycle (light on from 7:00 AM to 7:00 PM) and given conventional food (normal chow diet [NCD]; CE7 or high fat diet [HFD]; HFD32; Clea, Osaka, Japan) and water ad libitum. The % kcal from each ingredient was as follows. NCD – protein 20.5%, fat 10.1%; and HFD – protein 20.1%, fat 56.7%.

Experimental procedures and care of animals were carried out according to the Fukushima Medical University Institute of Animal Care and Use Committee.

### Measurement of energy intake and BW

The juvenile and adult rats were each divided into two groups; an NCD group and a HFD group. The groups were fed their respective diets for 63 days, and food intake and BW were measured once every 3 days, at 5:00 PM. After 63 days, the groups were given their diets until measurement of fat mass, glucose tolerance test, and insulin tolerance test were completed (up to 72 days).

### Glucose tolerance test (GTT) and insulin tolerance test (ITT)

Glucose tolerance test and insulin tolerance test were performed at days 69 and 72, respectively, after starting NCD or HFD. In order to prevent effect of GTT on energy intake and BW, animals were given 3 days interval between GTT and ITT. Thus, ITT was performed at day 72. At this point, the rats were 13 weeks old (Juvenile group) and 18 weeks old (Adult group). On the test day, the animals were deprived of food starting at 9:00 AM. Then, at 2:00 PM, the rats were intraperitoneally injected with 2 g/kg/10 ml glucose (Otsuka Pharmaceuticals, Tokyo, Japan) or 1 IU/kg insulin (Humulin R, Eli Lilly Japan, Kobe, Japan). Blood were collected from cut tip of tail and glucose levels were measured by glucose meter (Glutest every, Arkray, Kyoto, Japan) at 0 min (before injection), as well as at 15 min, 30 min, 60 min and 120 min after glucose or insulin injection.

### Measurement of insulin levels

Level of insulin secretion during GTT were examined. On the day of experiment, the animals were deprived of food at 9:00 AM, Then, rats were intraperitoneally injected with 2 g / kg / 10 ml glucose (Otsuka Pharmaceuticals, Tokyo, Japan) at 14:00 PM. Blood samples (approximately 50 μl) were collected cut tip of tail before (0 min) and 30 min, 60 min, and 120 min after the glucose injection. Blood samples were centrifuged at 4 °C at 4000 rpm for 10 min. After centrifugation, plasma samples were collected and stock at − 80 °C until the day of insulin measurement assay.

Plasma insulin was measured by using insulin ELISA kit (Morinaga Ultra Sensitive Mouse/Rat insulin ELISA Kit, Morinaga Institute of Biological Science, Yokohama, Japan). Intra-assay and inter-assay precision was, C.V. ≤ 10% respectively.

### Measurement of muscle, visceral fat and subcutaneous fat

At 66 days after starting NCD or HFD, computed tomography (CT) imaging using a La Theta LCT-200 system (Hitachi Aloka Medical, Mitaka, Tokyo, Japan) was used for the measurement of muscle, as well as visceral and subcutaneous fat mass, as described previously [11]. Holders with inner diameters of 120 mm was used. Animals were scanned under isoflurane anesthesia. Anesthesia was induced in a small acrylic box using a flow of 500 ml/min O_2_ with 5% isoflurane and maintained in the scanner via a nose cone providing 200 ml/min 2.0% isoflurane. CT scans were performed from 1st lumbar spine (L1) to 4th sacral vertebra (S4). At this point, the rats were 12 weeks old (Juvenile group) and 17 weeks old (Adult group).

### Statistical analysis

All data were presented as mean ± SEM. The analysis of difference between time and treatment factors in BW and energy intake over 63 days, change of blood glucose levels in GTT and ITT experiment between NCD and HD groups were performed by two-way ANOVA followed by Tukey’s multiple range test. Student’s t-test was used for two-group comparisons: the comparison of total fat (%), subcutaneous fat mass, visceral fat mass, muscle mass, and muscle/BW (%) from the CT scanning, and plasma insulin levels in each time point. *P* < 0.05 was considered as significant. All statistical test were 2-tailed with 0.05 as the threshold level of significance.

## Results

### The effect of HFD on the energy intake and BW in the juvenile and adult rats

**Fig. 1:**
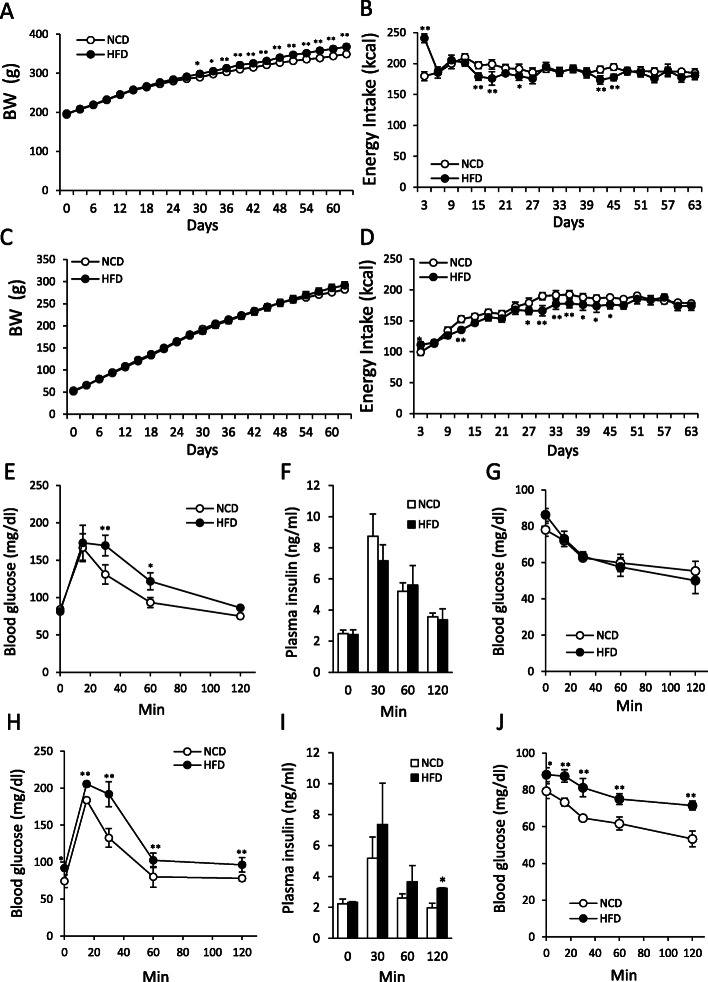
Changes in BW, energy intake and glucose metabolism in HFD- or NCD-fed adult and juvenile rats. **a**, **b**: Changes in BW **a** and energy intake **b** in the Adult group (*n* = 8, 8). **c, d**: Changes in BW **c** and energy intake **d** in the Juvenile group (*n* = 8, 8).**e**: Blood glucose levels after glucose tolerance test (GTT: 2 g/kg) in the Adult group (*n* = 4, 4). **f**: Plasma insulin levels after glucose injection (2 g/kg) at 0, 30, 60,120 min in Adult group (*n* = 4, 4). **g**: Blood glucose levels after insulin tolerance test (ITT: 1 IU/kg) in the Adult group. (*n* = 4, 4) H: Blood glucose levels after GTT (2 g/kg) in the Juvenile group (*n* = 4, 4). **i**: Plasma insulin levels after glucose injection (2 g / kg) at 0, 30, 60,120 min in the Juvenile group (*n* = 4, 3). **j**: Blood glucose levels after ITT (1 IU/kg) in the juvenile rats (*n* = 4, 4). GTT and ITT were performed at 69 and 72 days from onset of HFD feeding, respectively. Open circle and closed circle indicate normal chow diet (NCD) and high fat diet (HFD) feeding, respectively. **a-d, e, g, h, j,** * *P* < 0.05, ** *P* < 0.01. Two-way ANOVA, Tukey’s multiple range test. **f**, **i**, * *P* < 0.05, unpaired t-test

In the Adult group, HFD feeding significantly increased BW (Fig. 1a; F_1, 294_ = 122.14, *P* < 0.01) and energy intake (Fig. 1b; F_1, 280_ = 5.59, *P* < 0.05). However, there were no significant differences in total energy intake between the NCD and HFD groups (NCD; 3997.0 ± 123.8 kcal, HFD; 3934.6 ± 105.8 kcal). Especially during the first 3 days after starting HFD, energy intake was dramatically increased (Fig. 1b). However, in the Juvenile group, although energy intake for first 3 days showed a slight increase (F_1, 280_ = 42.7, *P* < 0.01), the energy intake throughout the experimental period was almost the same as that of the NCD group (Fig. 1d). There were no significant differences in total energy intake (NCD; 3566.2 ± 87.3 kcal, HFD; 3402.8 ± 112.6 kcal) and BW (Fig. 1c, F_1, 294_ = 0.04, *P* > 0.05) between the NCD and HFD groups during measurement.

### The effect of HFD on the glucose and insulin tolerance in juvenile and adult rats

In the Adult rats, blood glucose in the HFD group was significantly higher at 30 and 60 min after glucose injection compared with the NCD group (Fig. 1e, F_1, 24_ = 6.18, *P* < 0.05). There were no significant differences in insulin levels at any time points of GTT between NCD and HFD group (Fig. 1f). In the ITT, there were no significant differences in blood glucose between the NCD and HFD groups (Fig. 1g, F_1, 24_ = 0.06, *P* > 0.05).

The Juvenile group, the HFD group showed significantly higher blood glucose in both GTT and ITT in all time points (F_1, 24_ = 27.13, *P* < 0.01), including the blood glucose level before injection (t = 0 min) (Fig. 1h, j). Plasma insulin levels were tended to be increased at 30 and 60 min after glucose injection, and significantly increased at 120 min after glucose injection (Fig. 1i).

These data suggest that HFD feeding approximately for 2 months evoked glucose intolerance without affecting basal glucose level in the Adult group, whereas it evoked both increase of basal glucose level and decrease of insulin sensitivity in the Juvenile group.

### The effect of HFD on muscle, visceral fat and subcutaneous fat

**Fig. 2:**
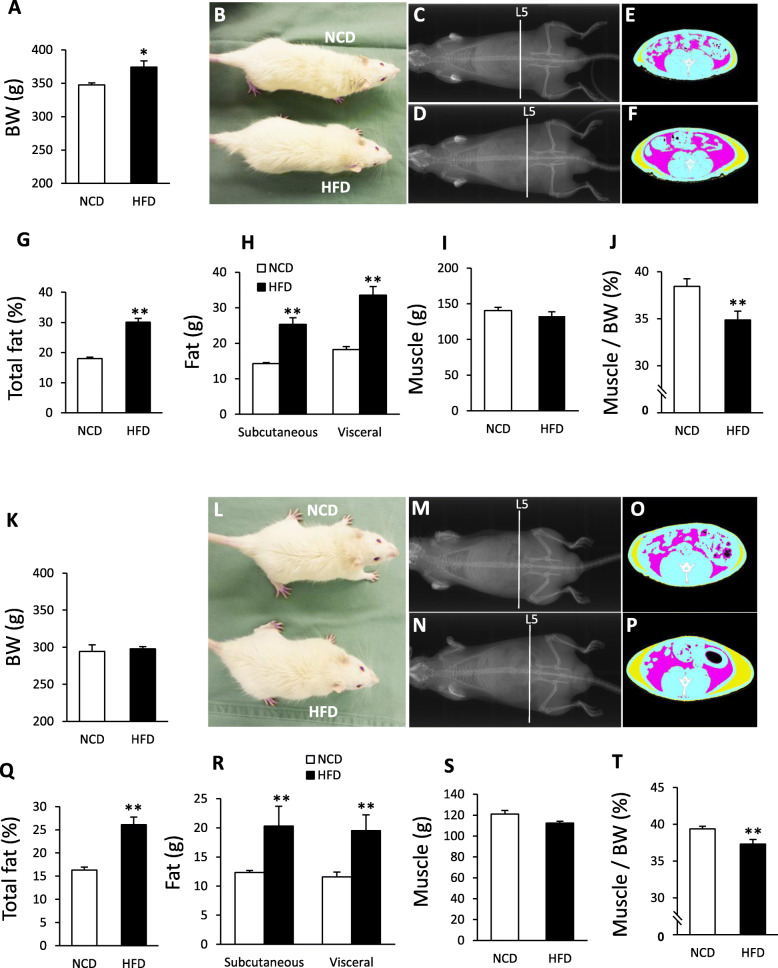
The comparison of body composition between NCD and HFD feeding in Adult and Juvenile groups. **a–j**: Adult group (18 weeks old) A: BW at 66 days of NCD and HFD feeding (*n* = 4, 4). **b**: Representative image of NCD and HFD fed rats. **c-f**: Representative X-ray **c, d** and CT images **e, f** of NCD **c, e** and HFD **d, f** fed rats. CT images of E and F are sectional images of the white dotted lines (level of vertebra L5) in C and D, respectively. Yellow areas indicate subcutaneous fat, and pink areas indicate visceral fat. The BW of NCD and HFD fed rats scanned by CT (corresponding to the rats in Fig. B) was 336.7 g and 372.5 g, respectively. **g**: The percentage of total fat to BW (*n* = 4, 4). **f**: Subcutaneous and visceral fat weight in the NCD and HFD fed rats (*n* = 4, 4). I: Muscle weight in the NCD and HFD fed rats (*n* = 4, 4). **j**: The percentage of muscle weight to BW in the NCD and HFD fed rats (*n* = 4, 4). **k–t**: Juvenile group (13 weeks old). K: BW at 66 days of NCD or HFD feeding (*n* = 4, 4). **l**: Representative image of rats fed NCD and HFD. M-P: Representative X-ray **m, n** and CT images **o, p** of NCD **m, o** and HFD **n, p** fed rats. CT images of O and P are sectional images of the white dotted lines (level of vertebra L5) in M and N, respectively. Yellow areas indicate subcutaneous fat, and pink areas indicate visceral fat. The BW of NCD and HFD fed rats scanned by CT (corresponding to the rats in Fig. L) was 292.3 g and 284.3 g, respectively. **q** The percentage of total fat to BW (*n* = 4, 4). **r**: Subcutaneous and visceral fat weight in the NCD and HFD fed rats (*n* = 4, 4). S: Muscle weight in the NCD and HFD fed rats. (*n* = 4, 4) T: The percentage of muscle weight to BW in the NCD and HFD fed rats (*n* = 4, 4). * *P* < 0.05, ** *P* < 0.01. unpaired t-test

In the Adult rats, the BW of the HFD group was significantly increased after 66 days HFD feeding (Fig. 2a). As shown in Fig. 2b, the appearance of the HFD fed rats was slightly bigger than that of the NCD fed rats. In addition, prominent visceral fat (pink area) and subcutaneous fat (yellow area) accumulation was observed in the HFD fed rats (Fig. 2c-f) with the percentage of total fat being 30.1 ± 0.4%, which was significantly higher when compared with the NCD group (18.0 ± 0.4%) (Fig. 2g). Subcutaneous fat and visceral fat in NCD fed rats were 14.3 ± 0.3 g and 18.2 ± 0.8 g, respectively. However, subcutaneous fat and visceral fat in HFD fed rats were 25.3 ± 1.8 g and 33.5 ± 2.4 g, respectively. Thus, both subcutaneous and visceral fat (total fat mass of mesentery fat, perirenal fat and epididymal fat) were 1.7 and 1.8 fold higher, respectively in the HFD group compared with the NCD group (Fig. 2h). The muscle mass in NCD and HFD fed rats were 140.5 ± 4.5 g and 132.6 ± 6.2 g, respectively. The muscle mass was slightly, but not significantly, lower (*P* = 0.09), although the percentage of muscle mass to the BW was significantly lower in the HFD group (NCD: 38.5 ± 0.8%, HFD: 34.9 ± 0.9%) (Fig. 2i, j).

In the Juvenile group, there were no significant differences in BW between the NCD and HFD groups, even after 66 days of HFD feeding (Fig. 2k). As shown in Fig. 2l, the appearance of the HFD fed rats was almost the same as that of the NCD fed rats. However, larger fat accumulation was observed in the HFD fed rats (Fig. 2m-p) with the percentage of total fat being 26.1 ± 1.6%, which was significantly higher compared with the NCD group (16.3 ± 0.6%) (Fig. 2q). Subcutaneous fat and visceral fat in NCD fed rats were 12.3 ± 0.4 g and 11.6 ± 0.8 g, respectively. However, subcutaneous fat and visceral fat in HFD fed rats were 20.3 ± 1.8 g and 19.5 ± 1.3 g, respectively. Both subcutaneous and visceral fat were significantly higher in HFD group (1.7 fold) compared with the NCD group (Fig. 2r). The muscle mass in NCD and HFD fed rats were 121.0 ± 3.4 g and 112.5 ± 1.5 g, respectively. The muscle mass was slightly, but not significantly (*P* = 0.06), lower in the HFD group (Fig. 2s). However, the percentage of muscle mass to BW was significantly lower in the HFD group (NCD: 39.4 ± 0.3%, HFD: 37.3 ± 0.6%) (Fig. 2t).

These data suggest that fat accumulation occurred in the HFD-fed Juvenile group without increasing BW.

## Discussion

Analysis of the effects of HFD on Juvenile and Adult group revealed that early age intervention of HFD induces normal weight obesity. Although HFD induced higher BW gain in the Adult group, with higher energy intake, it did not increase energy intake or BW more in the Juvenile group than in the HFD rats. However, in the HFD fed Juvenile group, higher increases in total fat, visceral fat, subcutaneous fat, insulin secretion, fasting blood glucose level were observed without change in BW. This condition is considered as normal weight obesity. Five months HFD exposure to 4 week juvenile mice also showed no change in BW compared with control diet fed mice [12]. Thus, this phenomena is considered to be common between rats and mice. In this study, we focused only on male rats. However, it is reported that morbidity of normal weight obesity is significantly higher in female than in male in human [13, 14]. Further study of sex differences and the effect of HFD exposure to female juvenile rats are required.

As for the energy intake, the HFD adult group showed higher energy intake from the beginning of HFD feeding. This corresponds to the previous studies showing energy intake being significantly higher in HFD fed rats, compared NCD fed rats for the first five to 6 days and then decrease [7, 15]. HFD is a highly palatable food and known to stimulate reward related area, such as ventral tegmental area (VTA) of the brain. The neurons in VTA is composed of dopamine neurons (~ 65%) and GABA neurons (~ 30%) [16–19]. VTA dopamine neurons are known as critical mediators in reward feeding [20], and the projection from dopamine neurons to the nucleus accumbens (NAc) promote the reward seeking and consumption [21, 22]. Because GABA neurons in VTA directly suppress the activity and excitability of neighboring dopamine neurons [16], GABA neurons also play a critical role for regulating reward related feeding. It can be considered that HFD feeding stimulated dopamine neurons and promoted reward related feeding when initiating HFD feeding in adult rats in this study.

However, this phenomenon, which HFD promote higher energy intake, was not observed in the Juvenile group and there were almost no differences in energy intake in the HFD and NCD fed juvenile rats. This is maybe due to the immaturity of GABAnergic neurons in VTA of juvenile rats. Our previous study revealed that the hyperactivity of dopamine neurons in VTA of juvenile rat compared with that of adults were induced by the undeveloped properties of GABA neurons which resulted in less inhibitory input to dopamine neurons [7]. This is considered as an underlying mechanism for juvenile rats to induce maximum energy intake regardless of HFD or NCD in order to promote growth [7].

However, even with the same caloric intake, HFD is known to induce significantly higher BW increase compared to NCD in adult rats [23]. Our previous study revealed that HFD feeding increases locomotor activity via undeveloped properties of GABA neurons in VTA and decreases inhibitory input to dopamine neurons leading to hyperactivity of dopamine neurons in the VTA of juvenile rats [7]. Thus, fact that there were no differences in BW between HFD and NCD fed juvenile rats in the current study may be explained by increasing energy expenditure following increased locomotor activity.

In the present study, blood glucose analysis revealed marked increase in basal blood glucose level with glucose intolerance, insulin secretion as well as reduced insulin sensitivity, in the HFD fed Juvenile group. Because the HFD fed Adult group showed only glucose intolerance but did not show increase in basal blood glucose and insulin secretion (indicating reduced insulin sensitivity), it is rational to consider that the impact of HFD-feeding for 72 days on blood glucose metabolism was larger in the Juvenile group than in the Adult group. The previous report using mice showed early exposure of HFD induced reduced phosphorylation of Akt in liver, which plays critical role in insulin signaling, increased fasting insulin level and pancreatic insulin content, without changing BW compared to control diet fed mice [12]. Increased plasma insulin levels and decreased insulin sensitivity was observed only in Juvenile HFD group, but not in Adult HFD group in our GTT and ITT experiments. These results suggest that the promotion of insulin secretion, and insulin resistance occurred only in HFD Juvenile group. Insulin resistance in juvenile stage is considered as a critical problem for the growth of various organs and development of metabolic syndrome. Insulin resistance is an important link between childhood obesity and cardiovascular risk [24]. This maybe the reason to induce more severe metabolic disturbance in normal weight obesity. Thus, improvement of insulin resistance may be important for the treatment of normal weight obesity.

Furthermore, fat accumulation was the same degree in the HFD fed adult (1.7–1.8 fold compared to NCD fed adult) and HFD fed Juvenile (1.7 fold compared to NCD fed juvenile) group. However, an increase in insulin secretion, fasting glucose level and decrease in insulin sensitivity was only observed in the HFD fed Juvenile group. These data indicate the metabolic vulnerability to HFD-induced fat accumulation in the juvenile rats. HFD-feeding induces lipotoxity and metabolic inflammation in various organs, such as the brain, muscle and pancreas [25]. These tissues of juvenile animals are considered to be in the developmental stage and are therefore still immature [7, 26, 27]. This may explain why a larger impact of glucose metabolism has been found in developing juvenile rats than matured adult rats.

The present data suggest that HFD feeding from early life may induce normal weight obesity after maturation. This is consistent with a previous study which also reported that early HFD feeding did not result in significant weight gain, and increased epididymal and retroperitoneal adipose tissue [28]. Recently, it has been reported that, in addition to overweight obesity, normal weight obesity has also been associated with metabolic disorders, and increased risk of metabolic syndrome and cardiovascular disease [5, 29]. In addition, normal weight obesity patients had significantly higher levels of inflammatory cytokines and higher IL-6 concentrations than those in non-obese and obese patients [30]. Moreover, normal weight obesity patients are at a significantly increased risk for cardiovascular events and mortality than non-obese and obese patients [30].

At present, obesity is evaluated only with BMI, due to its simplicity and validation in diagnosis. However, BMI is not an ideal index to evaluate obesity since it is calculated using only height and BW; thus, it is difficult to distinguish fat from lean mass. A previous study suggested the importance of using direct measurement of adiposity [30]. The current study showed that in spite of being normal weight, increased visceral fat, fasting glucose, insulin secretion and glucose intolerance were observed when rats were fed with HFD starting from 3 weeks old. The present study clearly shows the importance of considering fat and lean mass composition for evaluation of obesity and for the promotion of health.

Considering the health risk of having normal weight obesity, further studies are required. We believe that the normal weight obesity animal model shown in the present study is simple to generate and ideal for such studies.

In conclusion, our study provides support to the idea that high fat meals in critical growth periods may induce normal weight obesity in children, which will gradually develop into overweight obesity in adulthood. It emphasizes the importance of balanced meals starting in early life.

## Data Availability

The datasets used and analyzed during the current study available from the corresponding author on reasonable request.

## Abbreviations

ANOVA: Analysis of variance
BMI: Body mass index
BW: Body weight
CT: Computed tomography
GABA: Gamma-aminobutyric acid
GTT: Glucose tolerance test
HFD: High fat diet
ITT: Insulin tolerance test
NCD: Normal chow diet
SEM: Standard error of the mean
